# Triphenyltin isoselenocyanate: a novel nuclear retinoid X receptor ligand with antiproliferative and cytotoxic properties in cell lines derived from human breast cancer

**DOI:** 10.1007/s11010-023-04914-w

**Published:** 2024-01-16

**Authors:** Dana Macejova, Jakub Kollar, Pavel Bobal, Jan Otevrel, Daniela Schuster, Julius Brtko

**Affiliations:** 1 Institute of Experimental Endocrinology, Biomedical Research Center, Slovak Academy of Sciences, Dúbravská cesta 9, 845 05 Bratislava, Slovak Republic; 2 Department of Pharmaceutical and Medicinal Chemistry, Institute of Pharmacy, Paracelsus Medical University, Strubergasse 21, 5020 Salzburg, Austria; 3 Department of Chemical Drugs, Faculty of Pharmacy, Masaryk University, Palackého třída 1946/1, 612 00 Brno, Czech Republic

**Keywords:** Triorganotin isoselenocyanates, Retinoid X receptor, Apoptosis, Breast cancer

## Abstract

Several commercially available triorganotin compounds were previously found to function as agonist ligands for nuclear retinoid X receptor (RXR) molecules. Triphenyltin isoselenocyanate (TPT-NCSe), a novel selenium atom containing a derivative of triorganotin origin, was found to represent a new cognate bioactive ligand for RXRs. TPT-NCSe displayed a concentration- and time-dependent decrease in the cell viability in both human breast carcinoma MCF-7 (estrogen receptor positive) and MDA‑MB‑231 (triple negative) cell lines. Reactive oxygen species levels generated in response to TPT-NCSe were significantly higher in both carcinoma cell lines treated with TPT-NCSe when compared to mock-treated samples. Treatment with 500 nM TPT-NCSe caused a decrease in SOD1 and increased SOD2 mRNA in MCF-7 cells. The levels of SOD2 mRNA were more increased following the treatment with TPT-NCSe along with 1 μM all-*trans* retinoic acid (AtRA) in MCF-7 cells. An increased superoxide dismutase SOD1 and SOD2 mRNA levels were also detected in combination treatment of 500 nM TPT-NCSe and 1 μM AtRA in TPT-NCSe-treated MDA-MB-231 cells. The data have also shown that TPT-NCSe induces apoptosis via a caspase cascade triggered by the mitochondrial apoptotic pathway. TPT-NCSe modulates the expression levels of apoptosis‑related proteins, Annexin A5, Bcl‑2 and BAX family proteins, and finally, it enhances the expression levels of its cognate nuclear receptor subtypes RXRalpha and RXRbeta.

## Introduction

Besides new organic compounds containing platinum, the compounds undergoing intensive research in anticancer treatment, novel organotin compounds, and predominantly triorganotin preparations may yield in development of novel and promising antitumor drugs with non-cross-resistance with organoplatinum preparations [1, 2].

A number of various triorganotin molecules were found to possess the capability to exert cytotoxic properties against a number of tumour cell lines [3–5] and have been gaining growing importance in oncology [6, 7]. Triorganotin compounds in tumour cells were confirmed to affect various mechanisms at the cellular and molecular level, as they are capable of activating processes involved in the apoptotic pathways, e.g., p53 tumour suppressor, caspases and the proteins of the Bcl-2 family and TRAIL receptor [2]. Several triorganotin compounds have been reported to bind to nuclear retinoid X receptors (RXR) and thus play a significant role as high-affinity RXR ligands [8, 9] and function as transcriptional activators of target genes through permissive or conditional RXR—“partner nuclear receptor” heterodimers [7]. Retinoid X receptors (RXRalpha, RXRbeta, and RXRgamma) are members of the nuclear receptor superfamily of ligand-inducible transcriptional regulators, which control the expression of a large number of genes [10, 11].

**Fig. 1:**
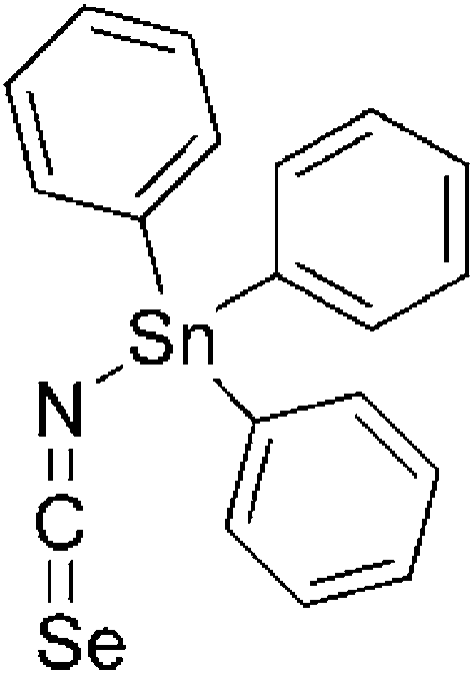
Triphenyltin isoselenocyanate (TPT-NCSe)

Recently, we reported in vitro antiproliferative and cytotoxic activities of a novel triorganotin compound, triphenyltin isoselenocyanate (TPT-NCSe) (Fig. 1), in human breast carcinoma MCF-7 (estrogen receptor positive) and MDA-MB-231 (triple negative) cell lines, the synthesis and analysis of which have been described precisely in our previous article [12]. The selenium atom containing a derivative of triorganotin compound caused higher cytotoxicity in MCF-7 cells compared to the MDA-MB-231 breast carcinoma cell line. DNA damage measurement has demonstrated crosslinks in both cell lines treated by increasing TPT-NCSe concentrations [12].

In this study, further biological analyses of the novel TPT-NCSe compound, in which two distinct chemical structures exerting antiproliferative actions (triorganotin and isoselenocyanate groups) have been merged into one molecule, were performed to investigate antiproliferative/cytotoxic potency and selected biological properties of this novel selenium atom containing compound. Since several triorganotin molecules are capable of interacting directly with the binding domain of the RXR molecule, the additional aims of this study were to find out (i) whether TPT-NCSe can affect the expression of nuclear retinoid and nuclear retinoid X expression in human breast cancer cell lines, (ii) whether TPT-NCSe can be considered to belong to a group of triorganotin compounds having properties of nuclear retinoid X receptor ligands and in the case it does, (iii) how triphenyltin isoselenocyanate binds in the binding pocket of retinoid X receptor.

## Materials and methods

### Reagents and antibodies

DMEM medium, phosphate‑buffered saline (PBS), dimethyl sulfoxide (DMSO), bovine serum albumin (BSA), BCA, 3-[4,5-dimethylthiazol-2-yl]-2,5-diphenyl tetrazolium bromide (MTT), and all-*trans* retinoic acid (AtRA) were obtained from Sigma-Adrich (MO, USA). FBS was obtained from HyClone Laboratories (GE Healthcare, Chicago, IL, USA).LDH Cytotoxicity WST Assay was acquired from Enzo Life Sciences (NY, USA), while Apo-ONE® Homogenous Caspase 3/7 Assay, RealTime-Glo™ Annexin V Apoptosis and Necrosis Assays were purchased from Promega (WI, USA). Minute™ Cytoplasmic and Nuclear Extraction Kit were obtained from Invent Biotechnologies, Inc. (MN, USA), Protease/phosphatase inhibitor cocktail from Cell Signaling Technology, Inc. (MA, USA), Blocking buffer from Li-Cor (NE, USA). Monarch® Total RNA Miniprep Kit was purchased from New England Biolabs (MA, USA), Thermo Scientific™ RevertAid H Minus First Strand cDNA Synthesis Kit from Thermo Fisher Scientific (MA, USA) and SensiFAST™ SYBR Lo‑Rox Kit from Bioline (UK).

Cellular Reactive Oxygen Species Detection Assay Kit (Red Fluorescence) was obtained from Abcam (Cambridge, UK) and Santa Cruz Biotechnology (TX, USA), and antibodies against B‑cell lymphoma 2 (Bcl‑2; dilution 1:100; cat. no. sc-7382), Bcl‑2‑associated X protein (Bax; dilution1:1000; cat. no. ab182733), Annexin V/ANXA5(dilution 1:1000; cat. no. ab54775), retinoid X receptor alpha (dilution1:1000; cat. no. ab125001), retinoid X receptor beta (dilution 1:500; cat. no. ab103027), retinoic acid receptor beta (dilution 1:1000; cat. no. ab124701), Beta-actin (dilution1:2500; cat. no. ab8227), Lamin B (dilution 1:500; cat. no. ab8982) and Anti-rabbit IgG (H + L) (DyLight™ 800 4X PEG Conjugate) and Anti-mouse IgG (H + L) (DyLight™ 680 Conjugate) secondary antibodies (dilution 1:15000; cat. no. #5151 and #5470, respectively) were purchased from Cell Signaling Technology (MA, USA). Triphenyltin isoselenocyanate (TPT-NCSe) was synthesised as previously described [12].

### Cells and treatment

The MCF-7 and MDA-MB-231 human breast carcinoma cell lines were purchased from the ACCT (American Type Culture Collection, VA, USA) and routinely cultured in a DMEM supplemented with 10% heat-inactivated FBS, 2 mM L-glutamine, 100 μg/mL penicillin, and 50 μg/mL streptomycin at 95% air condition with 5% of carbon dioxide (CO_2_) in 37 °C temperature. The cell lines were passaged weekly (approx. cell density of 1.0 × 10^6^cells/ml). Cells were plated in 96-well plates at 1‒2 × 10^4^cells/well density or in a Ø40mm Petri dish (TPP, Switzerland) at 1.2 × 10^6^cells/dish on the day before treatment, subsequently washed with PBS and exposed to various concentrations of TPT-NCSe for the time indicated. A stock solution of the tested compound was dissolved in ethanol. An equal volume of ethanol (final concentration < 0.02%) was added to the control cells.

### Cell viability assay

The TPT-NCSe derivative's effect on breast cancer cell survival was determined by MTT assay [13]. Cells were seeded at a 2 × 10^4^ cell density in 96-well culture plates and cultured for 24 h. Each concentration of the tested compound (added in the volume of 100 μL) was tested in quadruplicate. After 24 h, 48 h and 72 h, the cells were incubated with 10 μL of MTT (5 mg/mL in PBS) (Sigma-Adrich, MO, USA) and left in the dark at 37 °C in a CO_2_ incubator for an additional 3 h. After that, the medium was removed, the formazan crystals were dissolved in 100 μL of DMSO, and the absorbance was measured at 570 and 630 nm with a microplate reader (BioTek Instruments, Winooski, VT, USA). The concentration of drug that inhibited cell survival to 50% (IC_50_) was determined by an online tool “Quest Graph™ IC50 Calculator.” AAT Bioquest, Inc., 3 May. 2023, https://www.aatbio.com/tools/ic50-calculator. The results were given as the mean of three independent experiments.

### Membrane integrity

Cell membrane integrity of MCF-7 and MDA-MB-231 cells was analysed by determining the activity of lactate dehydrogenase (LDH) using LDH Cytotoxicity WST Assay (Enzo Life Sciences, NY, USA) according to the manufacturer’s protocol. Cells were seeded at a 2 × 10^4^ cell density in 96-well culture plates, cultured for 24 h, subsequently washed with PBS and exposed to various concentrations of TPT-NCSe (100 μL) for 24 h, 48 h and 72 h. 100 μL of LDH assay reaction mixture was added to each well. After 30 min incubation in the dark at room temperature, 50μL of Stop Solution was added. The optical density of the colour generated was determined at a wavelength of 490 nm using a microplate reader (BioTek Instruments, Winooski, VT, USA).

### Determination of reactive oxygen species

Intracellular reactive oxygen species (ROS) were measured using Cellular Reactive Oxygen Species Detection Assay Kit (Red Fluorescence) Abcam (Cambridge, UK) according to the manufacturer’s protocol. Cells were seeded onto 96-well plates at a density of 2 × 10^4^ cells per well and cultured for 24 h. After washing with PBS, a fresh medium containing TPT-NCSe (10, 100, 600 1000 and 10,000 nM) was added, and the cells were incubated for 3 h at 37 °C with 5% of carbon dioxide (CO_2_). Relative fluorescence intensity was determined with a microplate reader (BioTek Instruments, Winooski, VT, USA) at Ex/Em = 520/605 nm.

### Wound healing assay

The cell suspension was adjusted to a cell concentration of ca. 7 × 10^4^ cells/mL to obtain a confluent cell layer after 24 h. 70 µL of cell suspension was applied into each well of the Culture-Insert 2-Well in µ-Dish 35 mm, high (ibidi, 80206) and incubated cells at 37 °C and 5% CO_2_ for at least 24 h. After removing the Culture-Insert 2 Well with a sterile tweezer, the cell layer was washed with PBS to remove cell debris and non-attached cells. µ-Dish was filled with 2 mL cell-free medium with TPT-NCSe (10, 100 and 500 nM) or TPT-NCSe (100 and 500 nM) in combination with AtRA (1 μM). Removing the Culture-Insert 2-Well after appropriate cell attachment created a cell-free gap of approximately 500 µm. Microscopy (ZEISS AXIOVERT 40 CFL, Carl Zeiss AG Germany) was used to evaluate the wound healing process by measuring cell-covered area changes.

### Measurement of caspase 3/7 activity

Cells were seeded in 100 μL growth medium in white 96-well plates at the concentration of 1 × 10^4^ cells/well, cultured for 24 h, and subsequently washed with PBS. The caspase 3/7 activation was analysed by Apo-ONE® Homogenous Caspase 3/7 Assay (Promega, WI, USA) using fluorescent marker substrate (Z-DEVD-R110-DVED-Z) for detecting active caspase 3 and 7 was employed according to manufacturer’s instructions. Tested cells were incubated with TPT-NCSe (10, 100 nM, 1 μM and 10 μM), AtRA (1 μM) and with the combination of TPT-NCSe (100 and 10 μM) and AtRA (1 μM) in quadruplicates in a 96-well plate. The fluorescent signal was measured with a microplate reader (BioTek Instruments, Winooski, VT, USA) at Ex/Em = 485/528 nm. The fluorescent signal is proportional to the quantity of activated caspase. The results were given as the mean of three independent experiments.

### RealTime-Glo™ annexin V apoptosis and necrosis assay

Cells were seeded in 100 μL growth medium in white 96-well plates at 1 × 10^4^ cells/well, cultured for 24 h, and subsequently washed with PBS. The control wells without cells (growth medium only) were included to determine background luminescence and fluorescence. The appropriate dilution of TPT-NCSe was prepared in a growth medium at 4× the desired final concentration. A set of wells without a TPT-NCSe present was included. 100 μL of the TPT-NCSe dilution was added to the appropriate wells in the 96-well assay plate. Then, 100 μL of 2 × concentrated RealTime-Glo Annexin V Apoptosis and Necrosis Detection Reagent in the growth medium were added to each well. Cells were incubated in a covered 96-well assay plate at 37 °C/5% CO_2_ in a humidified cell culture incubator. Luminescence and fluorescence (Ex 475, Em 500–550) on the microplate reader (BioTek Instruments, Winooski, VT, USA) at 0–24 h were measured.

### Western blot analysis

The cells were seeded in a Ø40 mm Petri dish (TPP, Switzerland) at 1.2 × 10^6^ cells/well density, cultured for 24 h, and subsequently washed with PBS. After treatment with TPT-NCSe (10, 100 or 500 nM) or TPT-NCSe (100 or 500 nM) in combination with AtRA (1 μM) for 24 h, 48 h and 72 h, the cytoplasmic and nuclear protein fraction was isolated using Minute™ Cytoplasmic and Nuclear Extraction Kit (Invent Biotechnologies, Inc., MN, USA) containing a protease/phosphatase inhibitor cocktail (Cell Signaling Technology, Inc., MA, USA) according to the manufacturer’s instructions. The protein concentration was determined by the BCA method (Sigma‑Aldrich, USA). The exact protein amounts (20 μg for cytoplasmic and 30 μg for nuclear protein fraction in each lane) were loaded and separated by 10% SDS‑PAGE, then transferred onto polyvinylidene difluoride (PVDF) membranes. The membranes were blocked with Blocking buffer (Li-Cor, NE, USA) containing 0.05% Tween‑20 and then incubated with primary antibodies at 4˚C overnight. The next day, the PVDF membranes were washed three times in TBS‑T and incubated with fluorescently labelled secondary antibodies for 1 h at room temperature. The fluorescent signal of immunoreactive proteins was detected by imaging Odyssey system. Images were analysed with the Odyssey 2.0 analytical software (LI‐COR, NE, USA) based on optic densitometric analysis of the band. The proteins examined were normalised to the housekeeping protein human Beta-actin (cytosolic fraction) and human Lamin B1 (nuclear fraction).

### Reverse transcription‑semiquantitative real-time polymerase chain reaction

The cells were seeded in a Ø40 mm Petri dish (TPP, Switzerland) at 1.2 × 106 cells/well density, cultured for 24 h, and subsequently washed with PBS. After treatment with TPT-NCSe (10, 100 or 500 nM) or TPT-NCSe (100 or 500 nM) in combination with AtRA (1 μM) for 24 h, 48 h and 72 h, total RNA was isolated using Monarch® Total RNA Miniprep Kit (New England Biolabs, MA, USA) according to the manufacturer's instructions. The concentration of RNA was determined by spectrophotometry at 260 nm, and the purity was assessed using a NanoDrop 2000 spectrophotometer (Thermo Scientific, Germany). Reverse transcription (RT) was performed with 2 μg of total RNA and Thermo Scientific™ RevertAid H Minus First Strand cDNA Synthesis Kit (Thermo Fisher Scientific, MA, USA) according to the manufacturer's protocol. Semiquantitative real‑time PCR was performed in triplicates in a total volume of 20 μL using SensiFAST™ SYBR Lo‑Rox Kit (Bioline, UK) and 0.25 μM of each primer and RT product: 10 ng (RPS18) and 30 ng (for the other genes). Amplification and detection were performed with an ABI Prisma 7900HT detection system (Applied Biosystems, USA) under the following conditions: 95˚C for 2 min, 40 cycles of denaturation (95 °C, 5 s) and annealing (60 °C, 30 s), and the final melting curve analysis. The data are expressed using the 2^−ΔΔCq^ method as the relative level of each mRNA normalised to that of the housekeeping gene RPS18. The oligonucleotides of the primers employed in this study are summarised in Table 1 and elsewhere [14].

**Table 1:** Primers for semiquantitative real-time-PCR

Gene	Sequence
RARbeta2	5′-TTCAAGCAAGCCTCACATGTTTCCA-3′ 5′-AGGTAATTACACGCTCTGCACCTTTAG-3′
Caspase 3	5′-TACCAGTGGAGGCCGACTTC-3′ 5′-GCACAAAGCGACTGGTAGAAC-3′
Caspase 9	5′-TTCCCAGGTTTTGTTTCCTG-3′ 5′-CCTTTCACCGAAACAGCATT-3′
Annexin A5	5′-CAGTCTAGGTGCAGCTGCCG-3′ 5′-GGTGAAGCAGGACCAGACTGT-3′
Vimentin	5′-TCTCTGAGGCTGCCAACCG-3′ 5′-CGAAGGTGACGAGCCATTTCC-3′
SOD1	5′-TGAAGAGAGGCATGTTGGAGC-3′ 5′-TCTTCATTTCCACCTTTGCCG-3′
SOD2	5′-GGCCTACGTGAACAACCTGAA-3′ 5′-CTGTAACATCTCCCTTGGCCC-3′

### Molecular docking studies

To elucidate the potential binding mode of triphenyltin isoselenocyanate in RXR, we performed docking using Schrödinger small molecule drug discovery suite [15]. Several RXR crystal structures are available in the protein database [16]. Two of them were co-crystallized with triorganotin ligands, triphenyltin hydride (PDB entry code 3KWY [17]) and tributyltin hydride (PDB entry code 3E94 [18]). The structure 3KWY was used because of its structural similarity of its co-crystallized ligand to triphenyltin isoselenocyanate. After replacing the tin atom of the triphenyltin ligand with silicium, the 3D protein structure was prepared using the protein preparation wizard module in Schrödinger small molecule drug discovery suite (SMDD) [15, 19] using standard settings. Protonation states of the amino acids were adjusted to pH 7. Subsequently, the complex was gradually minimised by first minimising only hydrogens, then amino acid side chains and in the end the whole protein-ligand complex.

Since currently available force fields are not parameterised for the majority of heavy metal atoms, and the studied ligand TPT-NCSe contains both tin and selenium atoms, these needed to be replaced for the docking by silicon and sulphur, respectively. Atom replacement introduced bias in terms of atom size and partial charge. The partial charge bias was minimised by calculating atomic charges of TPT-NCSe quantum mechanically (QM) using density functional M06-2X with D3 correction in 6-31G**^++^ basis set [20] in water using Poisson Boltzmann solvation model [21].

Docking was performed using Glide module of Schrödinger SMDD suite [15, 22, 23] using the calculated QM charges in standard precision with ten output poses to report. Other settings were default. The docking protocol was tested for accuracy by redocking triphenyltin hydride into PDB structure 3KWY. The docked ligand showed a nice overlap with the co-crystallized ligand with an RMSD of 0.590 Å.

### Statistical analysis

Data are expressed as the means ± standard deviation (SD) of at least three independent experiments. The experimental procedures were performed in tri-/quadruplicate. Differences between more than two groups were assessed by one‑way analysis of variance followed by the respective post hoc tests using SigmaPlot® 11.0 (Systat Software GmbH).

## Results

### Cell viability assay-MTT and LDH release cytotoxicity

To evaluate the cytotoxic impact of a specific concentration, the IC_50_ concentration was determined. The compound under investigation exhibited a reduction in cell viability dependent on both the concentration and duration of exposure in both human breast carcinoma cell lines (as shown in Table 2).

**Table 2:** Cytotoxicity of triphenyltin isoselenocyanate in MCF-7 and MDA-MB-231 cells

IC_50_ [µM]	MCF-7	MDA-MB-231
24 h	1.109 ± 0.176	1.086 ± 0.126
48 h	0.447 ± 0.018	0.798 ± 0.063
72 h	0.49 ± 0.14	0.793 ± 0.011

The concentrations of agents that inhibited cell survival to 50% (IC_50_) at 24 h, 48 h and 72 h were measured by MTT assay and determined by an online tool “Quest Graph™ IC50 Calculator.” AAT Bioquest, Inc., 3 May. 2023, https://www.aatbio.com/tools/ic50-calculator. The results are expressed as the means ± standard deviation (SD) of three to four independent experiments

**Fig. 2:**
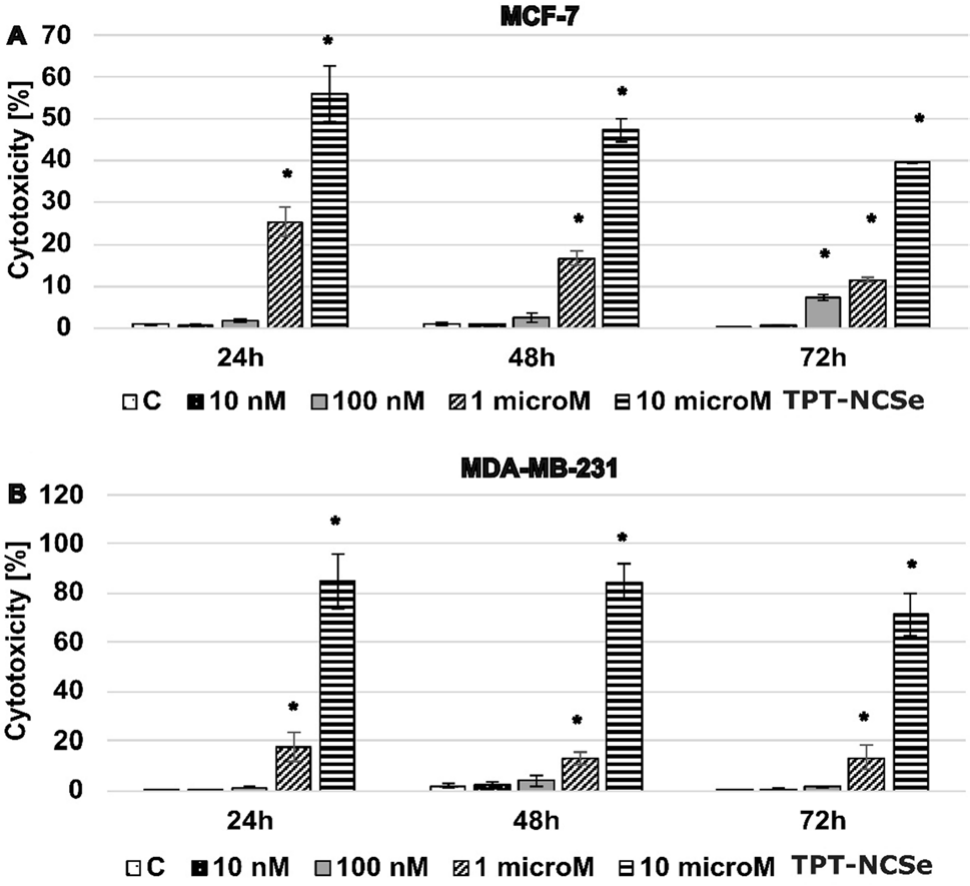
Cytotoxicity of TPT-NCSe in the MCF-7 (**A**) and MDA-MB-231 (**B**) cell. Cells were treated with indicated concentrations of tested substance for 24, 48, and 72 h, following colourimetric determination of LDH released into the culture medium. The results are expressed as the percentage of the LDH release in respect of lysis agent-treated control cells (100%). All values are represented as mean ± SD of three independent experiments. *p < 0.05 vs control

Lactate dehydrogenase (LDH) release into the culture medium was examined following 24 h, 48 h and 72 h exposure to TPT-NCSe. After quantification of the LDH leakage, we detected statistically significant differences in LDH release from MCF-7 (Fig. 2A) and MDA-MB-231 with the highest TPT-NCSe concentrations (1 and 10 μM). Increased concentration of TPT-NCSe resulted in a progressive cytotoxic effect. This was indicated by higher absorbance readings in the LDH assay, lower absorbance in the MTT assay, and a simultaneous decrease in the percentage of viable cells (data not displayed).

### Effect of TPT-NCSe in cellular reactive oxygen species

**Fig. 3:**
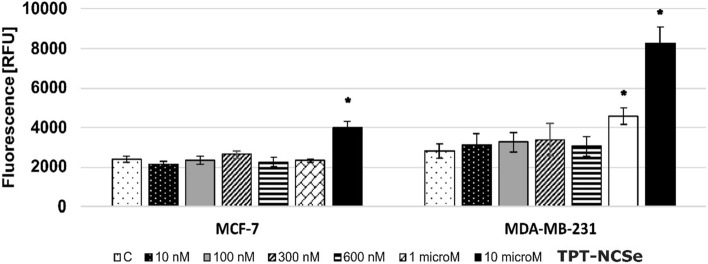
ROS generation in TPT-NCSe-treated MCF-7 (**A**) and MDA-MB-231 (**B**) cells. All values are represented as mean ± SD of three independent experiments. *p < 0.05 vs control

Based on the findings depicted in Fig. 3, the levels of reactive oxygen species (ROS) generated in MCF-7 (A) and MDA-MB-231 (B) cells treated with TPT-NCSe (10 µM) were significantly higher compared to the control group. These results suggest that cell death caused by the highest concentrations of TPT-NCSe is facilitated by the production of ROS, which can disrupt the cellular redox balance and potentially contribute to the mechanism of cell death.

**Fig. 4:**
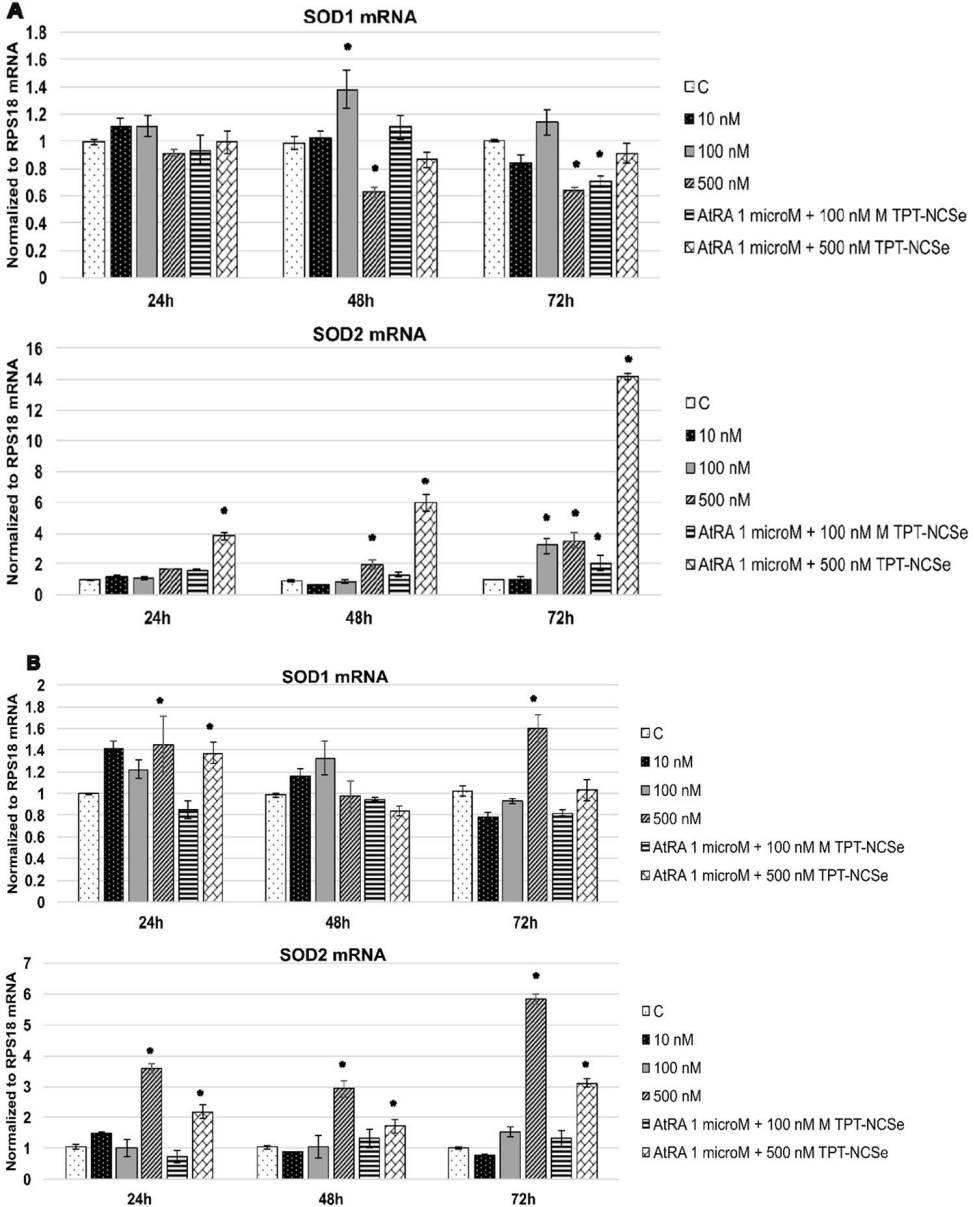
SOD1 and SOD2 mRNA levels in TPT-NCSe-treated MCF-7 (**A**) and MDA-MB-231 (**B**) cells. All values are represented as mean ± SD of three independent experiments. *p < 0.05 vs control

Superoxide dismutase (SOD) enzymes play a crucial role in regulating the levels of various reactive oxygen species (ROS) and reactive nitrogen species. By doing so, they effectively mitigate the potential toxicity of these molecules and maintain control over critical cellular processes that rely on their signalling functions. Treatment with 500 nM TPT-NCSe caused the combined decrease in SOD1 and increased SOD2 mRNA in MCF-7 cells (Fig. 4A). Moreover, the levels of SOD2 mRNA were more increased following the treatment with 500 nM TPT-NCSe along with 1 μM AtRA in MCF-7 cells. In TPT-NCSe-treated MDA-MB-231 cells, we have detected significantly increased SOD1 and SOD2 mRNA levels (500 nM TPT-NCSe and a combination of 500 nM TPT-NCSe and 1 μM AtRA).

### Wound healing assay

**Fig. 5:**
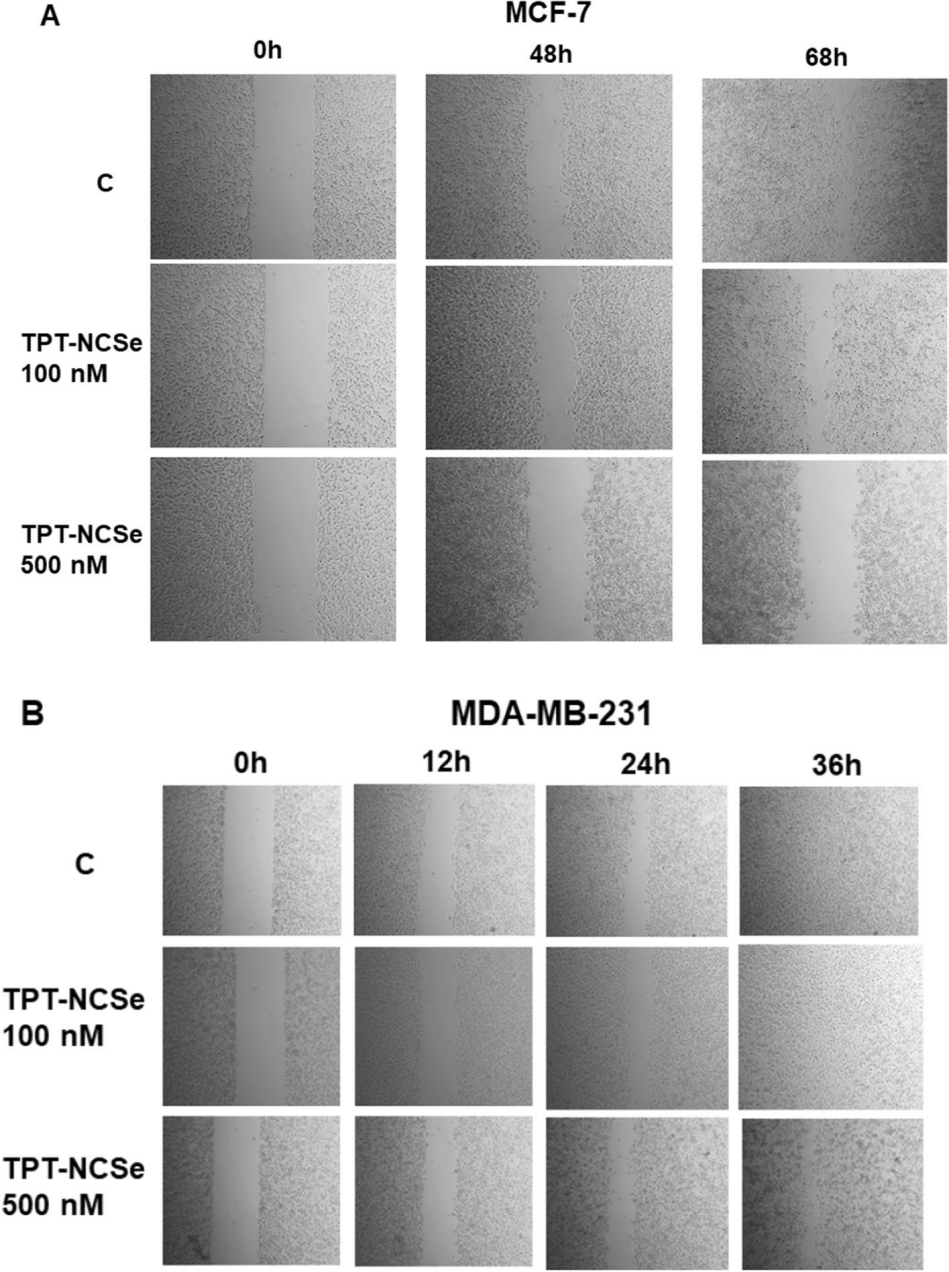
The inhibition of cellular proliferation and cell death in TPT-NCSe treated cells MCF-7 (**A**) and MDA-MB-231 cells (**B**)

The inhibition of cellular proliferation and cell death was observed in 500 nM TPT-NCSe treated MDA-MB-231 cells after 36 h (Fig. 5B) and in 100 and 500 nM TPT-NCSe treated MCF-7 cells after 48 h and 68 h (Fig. 5A). Both untreated and 10 nM TPT-NCSe-treated samples (data not shown) exhibited opposite patterns, where cell numbers at the wound site increased over time, leading to a reduction in wound gaps.

### Caspase 3/7 activation of TPT-NCSe-induced apoptosis

**Fig. 6:**
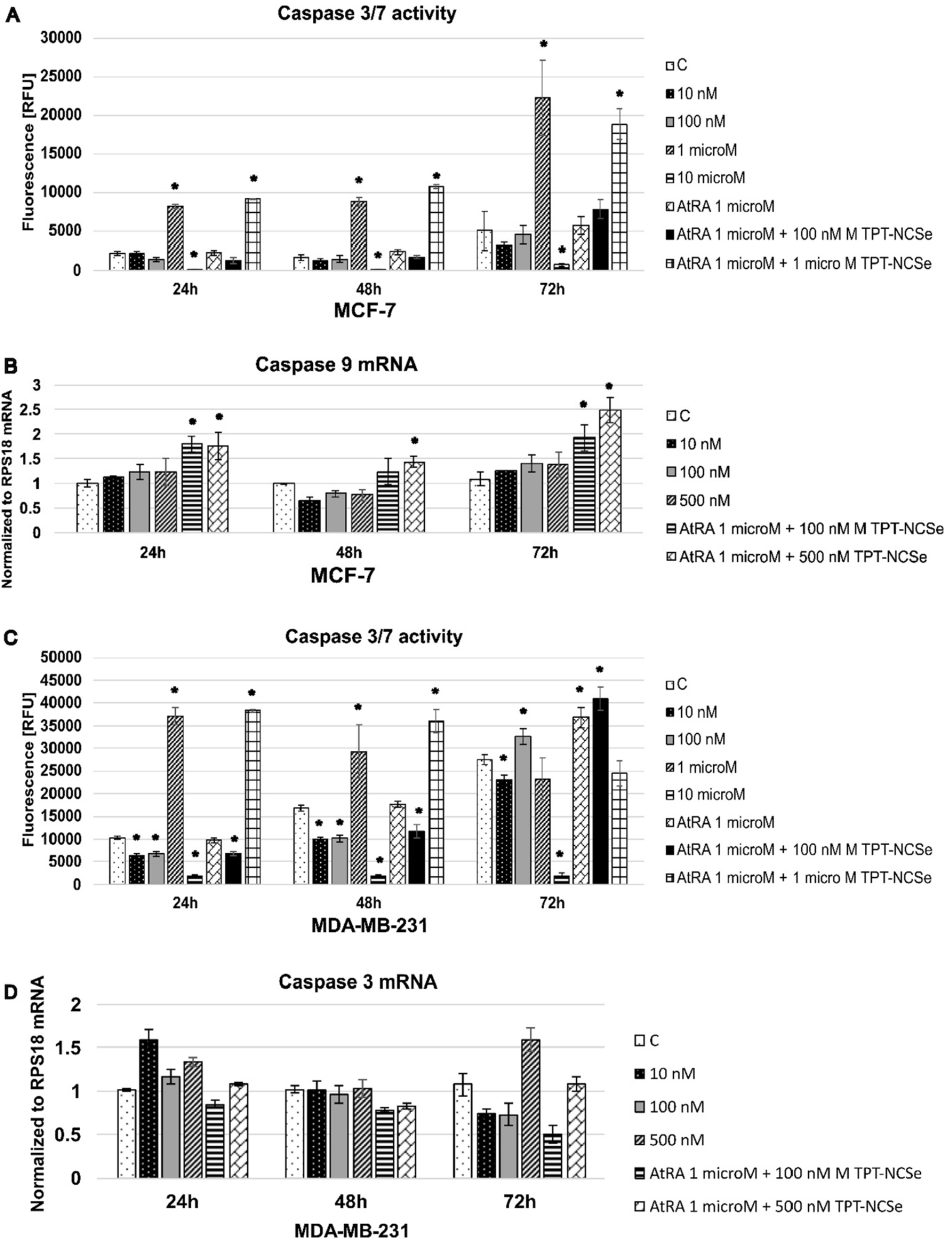
**A** Caspase 3/7 activity and **B** Caspase 9 mRNA levels in MCF-7 cells. **C** Caspase 3/7 activity and **D** Caspase 3 mRNA levels in MDA-MB-231 cells. The data are expressed as the mean ± SD of three independent experiments. MDA-MB-231 and MCF-7 cells were treated with TPT-NCSe for 24, 48 and 72 h. *p < 0.05 vs control

Activating the caspase 3 cascade is a critical component in multiple apoptotic pathways. To investigate the potential impact of TPT-NCSe on the apoptotic process, we evaluated the activity of caspase 3/7 in MDA-MB-231 and MCF-7 cells treated with TPT-NCSe. Figure 6 illustrates the observed elevation in caspase 3/7 activity during TPT-NCSe treatment. Specifically, treatment with 1 μM TPT-NCSe increased caspase 3/7 activity compared to untreated cells. Moreover, the levels of caspase 3/7 activity were more increased following the treatment with 1 μM TPT-NCSe along with 1 μM AtRA in MCF-7 and MDA-MB-231 cells (24 and 48 h) (Fig. 6A, C). Increased caspase 3/7 activation suggested that TPT-NCSe caused cell death through apoptosis. Since MCF-cells are caspase 3 deficient, the caspase-8 and caspase-9 mRNA were evaluated. Caspase-8 mRNA levels were increased after 24 h and decreased after 48 h TPT-NCSe (500 nM) treatment (data not shown). Caspase-9 mRNA in MCF-7 cells was decreased after 48 h treatment with TPT-NCSe and increased after 72 h (Fig. 6B).

In MDA-MB-231 cells, mRNA levels of caspase3 were increased after TPT-NCSe treatment for 24 h (10, 100 and 500 nM) and 72 h (500 nM) and decreased after 72 h (10 and 100 nM). Treatment with the combination of TPT-NCSe (100 nM) and AtRA (1 μM) caused a reduction of caspase 3 mRNA levels. (Fig. 6D). Moreover, we have found increased caspase-8 and 9 mRNA expression after 24 h and 48 h of treatment with TPT-NCSe 500 nM alone or in combination with AtRA (data not shown). The findings suggest that apoptosis induced by TPT-NCSe involves the activation of the caspase cascade and is initiated through the mitochondrial apoptotic pathway.

### Annexin V apoptosis/necrosis assay

**Fig. 7:**
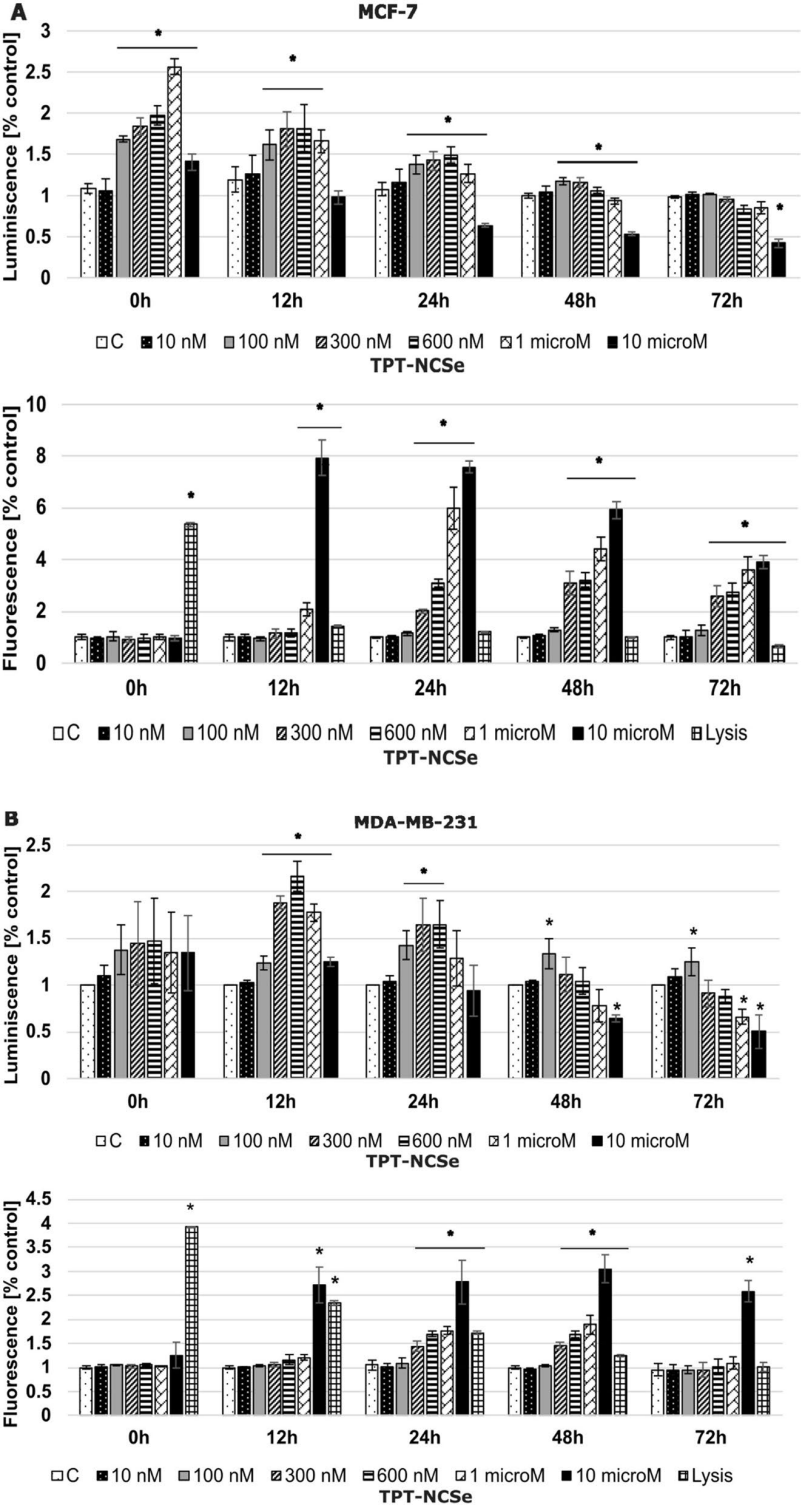
Cell apoptosis and necrosis determined by RealTime-Glo™ Annexin V Apoptosis and Necrosis Assay in response to the treatment with TPT-NCSe in MCF-7 (**A**) and MDA-MB-231 (**B**) cells. The data are expressed as the mean ± SD of three independent experiments. *p < 0.05 vs control

TPT-NCSe compound (300, 600 and 1000 nM)-induced apoptosis, as shown by the caspase 3/7 activation and Annexin V detection increase in both MCF-7 and MDA-MB-231 cells (Fig. 7). Annexin V detection increase was more prominent in the MDA-MB-231 cell line than in MCF 7 (12 h and 24 h).

### TPT-NCSe modulates the expression levels of apoptosis‑related proteins, annexin A5, Bcl‑2 and BAX family proteins

**Fig. 8:**
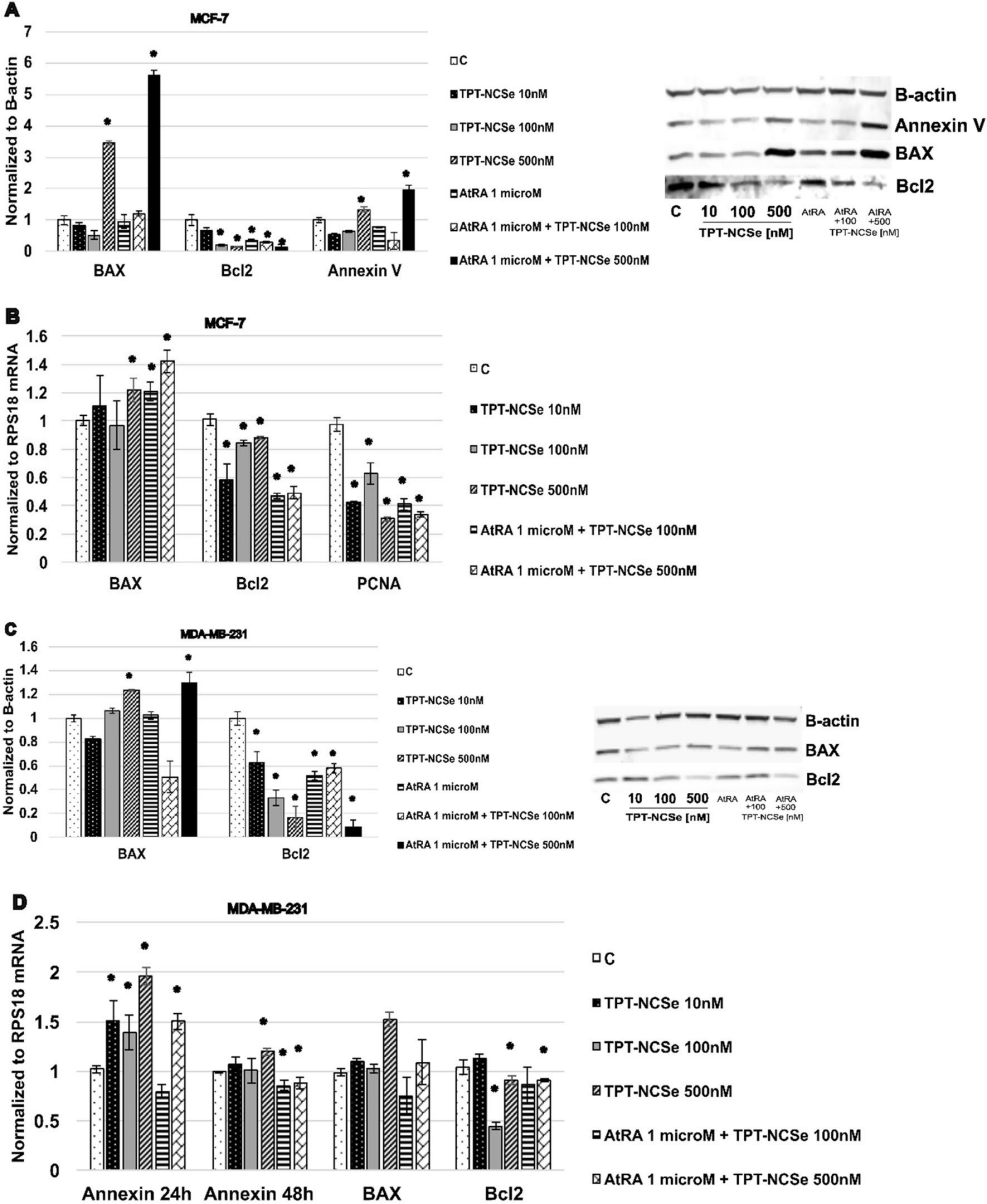
(**A**, **C**) Effect of TPT-NCSe on apoptosis‑related protein levels. The effect of TPT-NCSe on protein expression levels of Annexin A5, Bcl‑2 and BAX in MCF-7 and MDA-MB-231 cells was analysed by western blotting. Quantitative analysis was performed using Odyssey 2.0 analytical software. (**B**, **D**) The effect of TPT-NCSe on mRNA levels of Bcl‑2, BAX, PCNA and Annexin A5 in MCF-7 and MDA-MB-231 cells was analysed by semiquantitative real-time PCR. The results are expressed as the mean ± SD of three independent experiments. *p < 0.05 vs control

To investigate the mechanism underlying TPT-NCSe-induced apoptosis, Western blot analysis and real-time semiquantitative PCR (24 h, 48 h, 72 h) was employed to assess the expression levels of apoptosis-related proteins, specifically Bcl-2 and the pro-apoptotic protein BAX. The results revealed that in MCF-7 cells treated with TPT-NCSe (24 h, 48 h, 72 h), BAX protein levels were upregulated, while the expression of Bcl-2 was downregulated (Fig. 8A—data for 24 h and 72 h not shown). Moreover, the effect on BAX protein levels and Bcl2 protein and mRNA levels was more profound following the treatment with 500 nM TPT-NCSe and 1μMAtRA in MCF-7 cells for 48 h (Fig. 8A, B). The treatment with 500 nM TPT-NCSe and/or with 1 μM AtRA in MCF-7 cells for 24 h, 48 h and 72 h upregulated Annexin A5 protein (Fig. 8A– data for 24 h and 72 h not shown), however, no changes on mRNA levels (data not shown). These observations suggest that TPT-NCSe triggers the activation of the mitochondrial apoptotic pathway in MCF-7 cells by influencing the expression of Bcl-2 family proteins.

Similar effects were shown in MDA-MB-231 breast cancer cells. TPT-NCSe decreased Bcl‑2 after 24 h, 48 h and 72 h (Fig. 8C—data for 24 h and 72 h not shown) and increased BAX protein levels after 48 h treatment. Treatment with 1 μM AtRA+500 nM TPT-NCSe caused a further diminution of Bcl2 protein levels in MDA-MB-231 cells after 48 h treatment. The treatment with TPT-NCSe caused an upregulation of Annexin A5 mRNA levels in MDA-MB-231 cells (24 h and 48 h) and BAX mRNA (TPT-NCSe 500 nM for 24 h, 48 h and 72 h—data for 24 h and 72 h not shown), and downregulation of Bcl2 mRNA (TPT-NCSe 100 nM and 500 nM for 48 h and 72 h—data for 24 h and 72 h not shown) (Fig. 8D).

### Triphenyltin isoselenocyanate can bind in the RXRalpha binding pocket

**Fig. 9:**
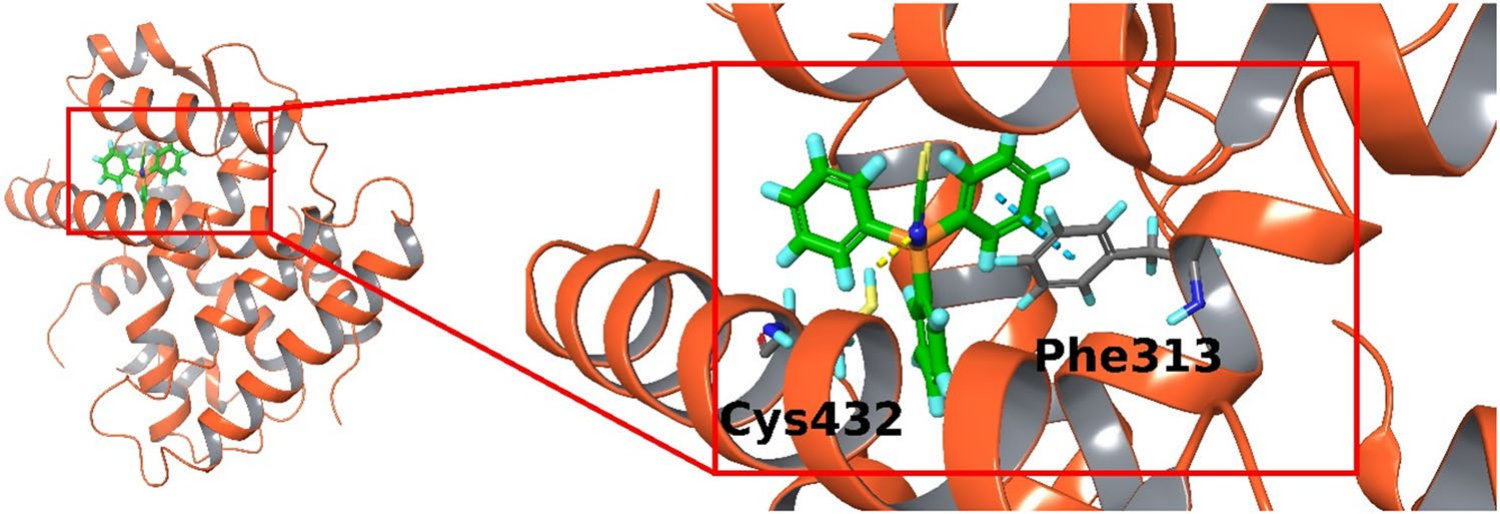
Docking mode of triphenyltin isoselenocyanate in RXRalpha binding pocket. One stacking interaction with Phe313 (dashed cyan line) and one hydrogen bond with Cys432 (dashed yellow line) are present

The predicted docking mode of triphenyltin isoselenocyanate is illustrated in Fig. 9. It shows stacking interaction between one ligand phenyl ring and Phe313 in RXR and a hydrogen bonding between the nitrogen of the isoselenocyanate group with the thiol group of Cys432 in RXR.

### TPT-NCSe modulates the expression levels of RXRalpha and RXRbeta nuclear receptors

**Fig. 10:**
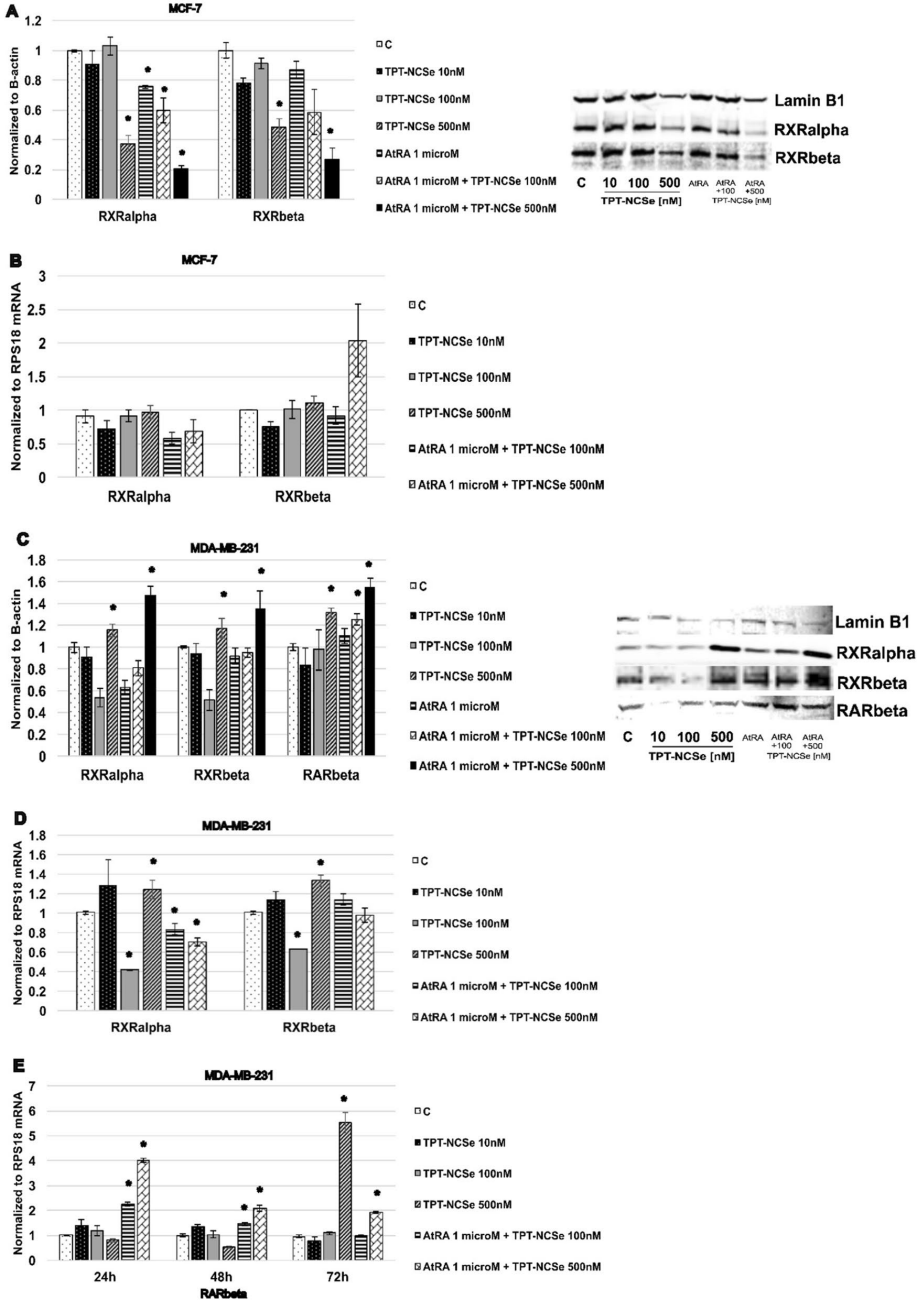
**A**, **C** Effect of TPT-NCSe on RXRalpha, RXRbeta, RARbeta2 protein levels. The effect of TPT-NCSe on protein expression levels in MCF-7 and MDA-MB-231 cells was analysed by western blotting. Quantitative analysis was performed using Odyssey 2.0 analytical software. **B**, **D**, **E** The effect of TPT-NCSe on protein expression and mRNA levels of RXRalpha, RXRbeta, and RARbeta2 in MCF-7 and MDA-MB-231 cells was analysed by semiquantitative real-time PCR. The results are expressed as the mean ± SD of three independent experiments. *p < 0.05 vs control

Western blot analysis and real-time semiquantitative PCR (48 h) were employed to assess the expression levels of protein expression and mRNA of both RXRalpha and RXRbeta in MCF-7 and MDA-MB-231 cell lines. In MCF-7 cells, treatment with TPT-NCSe (500 nM) downregulated protein expression of both RXRalpha and RXRbeta receptors (Fig. 10A), with no changes in mRNA levels (Fig. 10B). Downregulation of protein levels was more profound following the treatment with 500 nM TPT-NCSe in combination with RAR ligand 1 μM AtRA in MCF-7 cells (Fig. 10A).

TPT-NCSe (500 nM) increased mRNA and protein expression of both RXRalpha and RXRbeta in MDA-MB-231 cells (Fig. 10C, D). Concomitant treatment of MDA-MB-231 cells with AtRA and TPT-NCSe resulted in increased expression of RXRalpha, RXRbeta and RARbeta2 mRNA and protein levels (Fig. 10C, D, E).

## Discussion

New data on further biological properties of triphenyltin isoselenocyanate, a novel organic compound, in which two different molecule parts, triorganotin and isoselenocyanate, were merged into one molecule, enabling to generate a combined cytotoxic effect, represents a logical continuation of our recent work [12]. Moreover, we also bring novel and rather important information achieved from the in silico data showing that triphenyltin isoselenocyanate can represent nuclear retinoid X receptor ligand, which might play an important role in affecting biological effects mediated by “RXR—nuclear receptor” permissive or conditional heterodimers [10].

The MTT and LDH release cytotoxicity assay data showed a decrease in the viability of both MCF-7 and MDA-MB-231 cells after treatment with TPT-NCSe. The release of LDH into the extracellular medium due to membrane damage indicates lytic cell death. The findings from the study demonstrate that TPT-NCSe compromised cell membrane integrity in both MCF-7 and MDA-MB-231 cells in a manner that was dependent on the dosage of TPT-NCSe administered. TPT-NCSe-induced viability decrease of the MCF-7 cell line was significantly more pronounced than that in the MDA-MB-231 cell line. Extended periods of exposure to TPT-NCSe led to increased toxicity in the cells. The findings demonstrate a dose-dependent increase in TPT-NCSe toxicity. Furthermore, TPT-NCSe-induced LDH release in a concentration- and time-dependent manner, indicating that TPT-NCSe compromised the membrane potential of MCF-7 and MDA-MB-231 cells [12]. The IC_50_ results obtained from our study are comparable with our earlier report [12]. For further experiments, IC_50_ concentrations of TPT-NCSe at 48 h were used to demonstrate the effect of TPT-NCSe against MCF-7 and MDA-MB-231 cells.

Cells exposed to TPT-NCSe have the potential to respond through various mechanisms, including necrosis, where they experience loss of membrane integrity, or other forms of cell death, such as apoptosis. To assess whether breast cancer cells treated with varying concentrations of TPT-NCSe exhibited necrosis or apoptosis, the RealTime-Glo-Annexin V Apoptosis and Necrosis assay was utilised. The results obtained from this assay indicated that the apoptotic phenotype of cell death was observed in both human breast cancer cell lines treated with TPT-NCSe. Specifically, TPT-NCSe induced a significant increase in luminescence, indicating the binding of annexin V fusion protein to exposed phosphatidylserine (PS), a characteristic feature of apoptosis. Additionally, there was an increase in fluorescence, reflecting membrane integrity loss typically associated with secondary necrosis. This distinct kinetic difference in signal generation is a defining characteristic of the apoptotic phenotype [24].

The caspase 3 activation cascade is critical in various apoptotic mechanisms [25]. In order to study the potential impact of TPT-NCSe on the apoptotic pathway, we used a caspase 3/7 fluorescent assay to examine the activity of caspases in TPT-NCSe treated MCF-7 and MDA-MB-231 cells. 1 µM TPT-NCSe increased the activity of caspase 3/7 when compared to untreated cells. Moreover, the levels of caspase 3/7 activity were more increased following the treatment with 1 µM TPT-NCSe and 1 µM AtRA in MCF-7 cells. In the case of the highest concentration of 10 µM, we detected almost no caspase 3/7 activity after 24 h, 48 h and 72 h, which, in accordance with the significantly increased fluorescence signal of the necrosis assay, the results from the MTT assay and cytotoxicity assay and the very low signal in luminescence-annexin V fusion protein binding to exposed PS indicate that the cells underwent necrosis. However, MCF-7 cells are known to lack caspase 3 and might be insensitive to many chemotherapeutic agents. The difference of TPT-NCSe induced a significant increase in the binding of annexin V fusion protein to exposed phosphatidylserine (PS) in MCF-7 and MDA-MB-231 cells, indicating that MDA-MB-231 cells might respond more robustly to apoptosis-inducing signals of TBT-NCSe treatment.

The increase in caspase-8 and caspase-9 mRNA expression in MDA-MB-231 cells suggests that TPT-NCSe potentially induces apoptosis. Caspases-8 and 9 are initiator caspases that play essential roles in the intrinsic and extrinsic apoptotic pathways, respectively. Activation of caspase-8 and caspase-9 can trigger a cascade of downstream events that ultimately lead to the fragmentation of DNA and the breakdown of cellular components [26]. This process is often dysregulated in cancer cells, leading to their survival and proliferation. Therefore, the increase in caspase-8 and caspase-9 mRNA expression in MDA-MB-231 cells could potentially contribute to the inhibition of cancer cell growth and the induction of cell death. Thus, these findings suggest that apoptosis induced by TPT-NCSe involves the activation of the caspase cascade and is initiated through the mitochondrial apoptotic pathway.

Furthermore, our findings indicated that the mechanism of cell death in the tested breast cell lines may involve oxidative stress. Oxidative stress is a vital toxicity mechanism associated with exposure to compounds [27]. Since triorganotin compound can induce oxidative stress in breast cancer cells by inducing cellular ROS production, we measured ROS generation in both MCF-7 and MDA-MB-231 cells. The results obtained from our study suggest that cell death caused by the highest concentrations of TPT-NCSe is facilitated by the production of reactive oxygen species (ROS). This ROS production has the potential to disrupt the cellular redox balance, which could serve as a contributing factor to the mechanism of cell death. The generation of ROS mainly occurs during a decrease in the electron flow rate by high mitochondrial membrane potential (Δψm) [28, 29]. Our recent study demonstrated that TPT-NCSe treatment resulted in a concentration-dependent alteration of mitochondrial membrane potential in both cell lines. This was evidenced by the findings from JC-1 staining, indicating changes in the mitochondrial membrane potential induced by TPT-NCSe [12]. Mitochondrial damage is a common characteristic of both TNFalpha-induced caspase-dependent and/or—independent pathways and was found under excessive ROS production in MCF-7 cells [30]. Oxidative stress plays crucial roles in numerous normal biochemical processes, and any dysregulation in their functioning can lead to pathological conditions. The excessive production of reactive oxygen species (ROS) can activate various signalling pathways that initiate apoptosis, including the intrinsic and extrinsic pathways. Therefore, ROS generation has been established as a significant contributor to the process of apoptosis [30]. Our research data provide compelling evidence for a molecular mechanism by which TPT-NCSe induces ROS generation, suggesting that it could be one of the factors contributing to the induction of apoptosis.

SOD1 plays a role in scavenging ROS in the cytoplasm of cells, which can help protect cells from oxidative stress. SOD2 plays a similar role to SOD1, but in the mitochondria, which are the energy-producing organelles within cells. Studies have shown that SOD1 is overexpressed in various types of cancer, including breast cancer, and its overexpression has been associated with tumour growth, invasion, and metastasis [31]. Conversely, SOD2 is often downregulated in cancer cells, and its expression has been linked to tumour suppression [32].

TPT-NCSe (500 nM) decreases SOD1 mRNA, increases SOD2 mRNA in MCF-7 breast cancer cells, and increases SOD2 mRNA in MDA-MB-231 cells. Therefore, TPT-NCSe could potentially have a dual effect: reducing the levels of an enzyme that promotes cancer cell growth and increasing the levels of an enzyme that suppresses cancer cell growth. This could result in an overall inhibition of tumour growth. Moreover, the inhibition of cellular proliferation and cell death was observed in TPT-NCSe-treated MDA-MB-231 and MCF-7 cells. No relevant data are available in literature on the effect of AtRA, a cognate ligand of RARs, on the SOD1 or SOD2 expression in human breast cancer cell lines. On the other hand, it has been clearly demonstrated that AtRA markedly induces manganese superoxide dismutase (SOD2) activity in human neuroblastoma [33]. These observations on enhanced SOD2 activity by AtRA are in agreement with our SOD2 mRNA data on human breast cancer cells in the presence of 1 µM AtRA.

Western blot analysis and real-time PCR showed that TPT-NCSe increased Annexin A5 protein, BAX, and decreased Bcl‑2, both protein and mRNA levels in MCF-7 cells. Moreover, we have detected reduced PCNA mRNA levels and Nurr1 mRNA levels (data not shown). The decrease in PCNA mRNA indicates a reduction in cell proliferation or DNA damage, another cancer hallmark. The decrease in Nurr1 mRNA is significant as it is involved in the progression and survival of cancer cells [34]. The findings further supported the observation that treatment with TPT-NCSe resulted in anti-migratory and anti-proliferative effects. Therefore, the overall effect of TPT-NCSe is a decrease in cancer progression, and it could be a potential therapeutic agent for cancer treatment.

However, the results of our present study and our previous findings on triorganotin compounds binding to RXR nuclear receptors [9, 10, 35] enable us to suppose that TPT-NCSe could affect the viability of MCF-7 and MDA-MB-231 breast cancer cells by binding to RXR receptors. Molecular docking of TPT-NCSe produced an energetically favourable pose within the binding site of the RXR ligand binding domain, supporting our assumption that TPT-NCSe might induce its cellular effects by binding to RXR. However, docking results should be interpreted with caution. TPT-NCSe contains tin and selenium atoms, heavy atoms not parameterised within typical force fields suitable for proteins. Therefore, these had to be replaced by silicium and sulphur, respectively, which introduced bias. Even though this bias was partially accounted for using quantum mechanical charges on all ligand atoms, atoms sizes cannot match sulphur and selenium, and the charges of protein atoms were still enforced by OPLS3 force field and would not adapt.

In less than two decades, it has been clearly demonstrated that triorganotin compounds function as agonist ligands for nuclear retinoid X receptors [36]. Despite lacking structural similarities with known hormone ligands, triorganotins have been found to interact covalently with the cysteine residue (C432) of helix H11 located at the entrance to the ligand binding pocket of the RXR molecule. This unique interaction has been observed with both trialkyltin and triaryltin compounds [37]. Thus, tributyltin or triphenyltin compounds were found to act as potent high-affinity activators of RXRs [9, 36]. In general, RXRs serve as crucial heterodimeric partners for numerous nuclear receptor partners PPARs, PXR, CAR FXR, LXRs forming permissive heterodimers, VDR, nonpermissive heterodimer and RARs or TRs conditional heterodimers, and take part in orchestration of multiple hormonal signalling pathways [7, 18, 36]. Thus, inappropriate activation of RXRs can significantly disrupt the body's homeostatic hormonal regulation, causing widespread disturbances [35].

We have found that TPT-NCSe (500 nM) was capable of increasing mRNA and protein expression of both RXRalpha and RXRbeta in MDA-MB-231 cells. Concomitant treatment of MDA-MB-231 cells with AtRA and TPT-NCSe resulted in increased expression of RXRalpha, RXRbeta and RARbeta2 mRNA and protein levels.

RAR-RXR heterodimers can activate several genes that induce apoptosis in cancer cells. The induction of apoptosis by RAR-RXR heterodimers is a complex process involving the modulation of various genes (BAX, PUMA (p53 upregulated modulator of apoptosis), TRAIL (tumour necrosis factor-related apoptosis-inducing ligand), Fas (also known as CD95), Death-associated protein kinase 1 (DAPK1)) and signalling pathways, including the activation of caspases, the modulation of Bcl-2 family proteins, and the regulation of survival pathways such as the PI3K/AKT pathway [38–40].

In conclusion, our study indicates that TPT-NCSe might act via activation RXR-RAR heterodimer since an enhancement of induction of caspase 3/7 activity, SOD2 mRNA, Annexin A5 protein, BAX protein level and mRNA level, or inhibition in Bcl2 protein and mRNA levels in MCF-7 cells when treated along with AtRA, have been demonstrated. Last but not least, TPT-NCSe was also shown to enhance the expression of two retinoid X receptor subtypes at mRNA and protein levels.

## Data Availability

Enquiries about data availability should be directed to the authors.

## Abbreviations

Δψm: Mitochondrial membrane potential
AtRA: All-trans retinoic acid
DMSO: Dimethyl sulfoxide
LDH: Lactate dehydrogenase
MDA-MB-231: Estrogen-receptor-negative human breast cancer cell line
MCF-7: Estrogen-receptor-positive human breast cancer cell line
MTT: 3-[4,5-Dimethylthiazol-2-yl]-2,5-diphenyltetrazolium bromide
PBS: Phosphate-buffered saline
PS: Phosphatidylserine
PVDF: Polyvinylidene difluoride
QM: Quantum mechanical
RAR: Retinoic acid receptor
ROS: Reactive oxygen species
RXR: Retinoid X receptor
SMDD: Small molecule drug discovery
SOD: Superoxide dismutase
TPT-NCSe: Triphenyltin isoselenocyanate
TR: Thyroid hormone receptor
VDR: Vitamin D receptor
